# Actin in Herpesvirus Infection

**DOI:** 10.3390/v3040336

**Published:** 2011-04-12

**Authors:** Kari L. Roberts, Joel D. Baines

**Affiliations:** Department of Microbiology and Immunology, Cornell University, Ithaca, NY 14853, USA; E-Mail: klr54@cornell.edu

**Keywords:** herpes, actin, Rho GTPase, myosin

## Abstract

Actin is important for a variety of cellular processes, including uptake of extracellular material and intracellular transport. Several emerging lines of evidence indicate that herpesviruses exploit actin and actin-associated myosin motors for viral entry, intranuclear transport of capsids, and virion egress. The goal of this review is to explore these processes and to highlight potential future directions for this area of research.

## 1. Overview of Actin-Related Cytoskeletal Components

Actin is a highly dynamic protein critical for a wide variety of important cellular processes including cell division, adherence, migration, uptake of extracellular material, and intracellular transport of vesicles and other cargo. There are two basic forms of actin: monomeric or globular actin (G-actin) and filamentous actin (F-actin). Formation of F-actin begins by a nucleation event from proteinacious complexes such as actin-related protein 2/3 (Arp2/3) [1]. G-actin subunits assemble in a polarized fashion, forming an actin filament with distinctive ends. The plus or “barbed” end of a growing actin filament rapidly elongates while the minus or “pointed” end remains relatively static. This process is reversible causing actin filaments to rapidly depolymerize as subunits dissociate from the plus end. F-actin can also associate with other actin filaments to form more complex structures such as actin arrays or networks.

**Figure 1.**
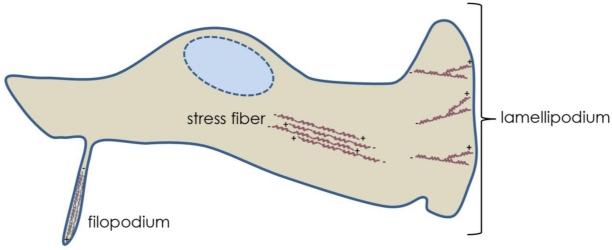
Examples of Actin Networks. Filopodium, stress fiber, and lamellipodium structures are featured showing actin polarities.

Three fundamental examples of actin networks are stress fibers, filopodia and lamellipodia (Figure 1). Actin stress fibers are short, contractile bundles of F-actin with alternating polarity. The filaments are held together through interactions with the dimeric bundling protein α-actinin and the motor protein myosin II, which enables contraction. These fibers are essential for maintaining cell attachment to the substratum and implementing changes in morphology. Filopodia contain stiff, tightly bound parallel actin bundles that polymerize toward the plasma membrane forming a thin, spike-like protrusion. These structures may act as probes or sensors of the extracellular environment and contain receptors for signaling as well as integrins and cadherins (cell adhesion molecules). Lamellipodia are highly branched networks of actin filaments, with the highest branching frequency occurring nearest the plasma membrane. This “dendritic” sheet-like array of F-actin comprises the leading edge of migrating cells and membrane ruffles. Generation of these actin structures requires the activity of Rho GTPases. These proteins are analogous to molecular switches, existing in either an active (GTP-bound) or inactive (GDP-bound) form. The GTPases associate with downstream effector proteins in signal transduction pathways [2,3]. The three most extensively characterized mammalian Rho GTPases, and those known to be involved in herpesvirus infections, are RhoA, Rac1 and Cdc42. RhoA is associated with the generation of actin stress fibers, Cdc42 with filopodia, and Rac1 with lamellipodia and ruffling.

Rho GTPases have been shown to be important for phagocytosis [3], a form of endocytosis involving the internalization of large particles at the cell surface, and known to be used by incoming herpesviruses (explained below). Initiation of phagocytosis involves receptor-mediated rearrangement of cortical actin localized near the position of the stimulating particle. During Fc receptor (FcR)-mediated phagocytosis F-actin accumulates beneath a pedestal-like formation and the particle is engulfed by extending pseudopodia that wrap around the particle and subsequently fuse [4]. In FcR-mediated phagocytosis in rat basophil leukemia mast cells, expression of dominant-negative Cdc42 results in the formation of pedestal-like structures beneath the particles but pseudopodia do not form, as observed by scanning electron microscopy (SEM) [5]. Expression of dominant-negative Rac1 results in particles enclosed within pseudopodia that do not fuse [5]. While FcR-mediated phagocytosis is known to require Cdc42 and Rac1, receptors for complement (CR)-mediated phagocytosis induce RhoA-dependent recruitment of Arp 2/3 to F-actin rich foci at the site of particle attachment. Although Arp 2/3 is involved in both types of phagocytosis, there are no pseudopod extensions in CR-mediated phagocytosis. Instead, the particle seems to sink into the cell and Cdc42 and Rac1 are dispensable for particle uptake, unlike the case of FcR-mediated phagocytosis.

Since actin is crucial to many cellular processes, including uptake and short-range intracellular transport (such as the movement of melanosomes towards the plasma membrane), it is perhaps not surprising that herpesviruses utilize host actin during infection.

## 2. Overview of Herpesvirus Replication

The family Herpesviridae can be further categorized into three subfamilies: alpha-, beta- and gamma-herpesvirinae, all of which establish life-long infections in their hosts. Herpes simplex virus type 1 (HSV-1), varicella-zoster virus (VZV) of humans, and pseudorabies virus (PrV) of swine are examples of alphaherpesviruses. Human and mouse cytomegalovirus (HCMV and MCMV, respectively) are commonly studied betaherpesviruses, and Epstein-Barr virus (EBV) and Kaposi’s sarcoma-associated herpesvirus (KSHV) are both gammaherpesviruses that infect humans. The virions of herpesviruses comprise a double stranded linear DNA genome contained within a proteinaceous capsid. A number of proteins surround the capsid and are collectively called the tegument. Surrounding the tegument is an envelope containing a number of viral glycoproteins.

Herpesviruses enter cells either by fusion at the plasma membrane or are endocytosed by the cell to eventually fuse within an intracellular vesicle [6–10]. Regardless of how the virus enters, once the capsid accesses the cytosol, viral tegument proteins dissociate from the capsid to prime the cell for infection while the capsid traffics toward the nucleus along microtubules using the molecular motor dynein [11–12]. Capsids migrate to and dock at nuclear pores where they subsequently release the viral genome through a unique capsid vertex (the portal) and the genome enters the nucleus through the nuclear pore [13–15]. Nuclear genomes are used as templates for viral transcription, which occurs in three temporal stages [16,17]. Nascent replicated genomes are then packaged into newly assembled capsids. The packaged capsids associate with the inner nuclear membrane and subsequently bud into the perinuclear space to acquire a primary envelope. This envelope is lost as the virion envelope fuses with the outer nuclear membrane and the nucleocapsid is released in the cytosol. Next, the capsid associates with tegument proteins as it traffics towards the *trans*-Golgi network (TGN). TGN-derived vesicles are believed to be the major site of secondary envelopment where the virus acquires its final mature envelope, which is embedded with viral glycoproteins [18]. Fusion of these transport vesicles with the plasma membrane releases virions into the extracellular environment.

## 3. Actin and Herpesvirus Entry

Incoming herpesvirions first encounter host actin during entry into cells (for another review on herpesvirus interactions with the cytoskeleton see reference [19]). The cortical actin lining the cytosolic side of the plasma membrane is a meshwork of F-actin connected to the cell surface through surface receptors. Various signaling pathways can alter the cortical actin. In the case of viral infection, virion glycoproteins bind to specific host cell receptors, ultimately causing the envelope and plasma membrane to fuse, delivering the capsid and tegument into the cytosol. Whereas glycoprotein D (gD) is an important receptor binding protein in most alphaherpesviruses except VZV, gB, gH and gL are all required for virion fusion [20–22].

Depending on the cell type, the fusion event required for HSV-1 entry occurs at a neutral pH at the plasma membrane (e.g., Hep2 and Vero cells) [10], through a pH-dependent endocytic pathway (e.g., HeLa cells and modified Chinese hamster ovary (CHO) cells expressing HSV-1 receptors) [8] or through a pH-independent endocytic pathway (C10 murine melanoma cells) [5]. Polymerized actin was shown to be dispensable for pH-independent entry of HSV-1 into Vero cells based on insensitivity to the actin depolymerizing drug cytochalasin D [10]. However, actin dynamics are required for the pH-dependent pathway utilized by HSV-1 in corneal fibroblasts (CF) and CHO cells, because HSV-1 entry was inhibited by cytochalasin D [7]. Thus, the dependence of actin during entry appears to be dictated by the pathway of entry and type of cell infected.

In actin-dependent entry, initial binding to the cell surface can influence cortical actin. For example, binding of HSV-1 gD to the surface receptor nectin-1 (a member of the immunoglobulin superfamily and also known as HveC and CD111) stimulates Rho GTPase signaling, which in turn can alter the morphology of cortical actin. In cell lines such as CF, Madin-Darby canine kidney II (MDCKII) and CHO cells, HSV-1 entry has been shown to induce Cdc42 signaling [24]. During entry into CHO cells expressing HSV-1 receptors, HSV-1 virions induce and attach to protrusions from the plasma membrane [7]. The presence of these protrusions correlates with RhoA activation. A model was proposed in which virions move along these protrusions (a process also known as “surfing”) to portions of the cell body for eventual entry in an actin-dependent phagocytosis-like uptake [7]. Phagocytosis and another form of endocytosis, known as macropinocytosis, have many similarities. These similarities include the requirement for actin, Rac1 and Cdc42. In contrast, phagocytic cells (e.g., macrophages) require RhoA in addition to Rac1 and Cdc42 [23]. Differently from the CHO cells, inhibition of RhoA activation did not block HSV-1 entry into MDCKII cells [24]. In these cells entry correlates with Cdc42 and Rac1 activation rather than RhoA [24]. As another example of cell specific roles of actin in entry, evidence indicates that HSV-1 utilizes a pH-dependent endocytotic pathway to infect keratinocytes [9]. Despite the fact that such pathways normally require activation of Rho GTPases, another study demonstrated HSV-1 entry into keratinocytes was insensitive to knock-down of Rac1, Cdc42 or RhoA. Therefore, entry in these cells may utilize a novel and, as of yet, unidentified entry pathway [25]. In contrast, the gamma herpesvirus KSHV gB mediates entry through association with the α_3_β_1_ integrin receptor and activates Rac1, Cdc42 and RhoA. As might be expected from activation of these Rho GTPases, KSHV induces the formation of actin stress fibers, filopodia and ruffling within 30 minutes post infection of human foreskin fibroblast cells [26].

PrV entry into sensory neurons of the trigeminal ganglion, which is an important step for the establishment of latent infections, induces the formation of varicosities (bulges or swellings along neuronal axons) or synaptic boutons [27]. Glycoprotein D was found to be both necessary and sufficient for formation of these virally-induced varicosities, and this formation was dependent upon Cdc42 but not Rac1 or RhoA [27]. Synaptic boutons have also been shown to be important egress sites for both PrV [27,28] and HSV-1 [29].

## 4. Herpesviruses and Nuclear Actin

Once internalized into the cell, the capsid must deliver the viral genome into the nucleus. Trafficking the sometimes considerable distance from the cell periphery to nucleus (axons of some sensory neurons are many centimeters in length) is mediated by microtubules and the molecular motor dynein [11–12]. As the cell begins transcribing viral genes and replicating the viral DNA, viral replication compartments form and the host chromatin is marginalized to the nuclear periphery. The HSV-1 infected nucleus also expands at about 8 hours post infection [30]. This nuclear expansion as well as marginalization of host chromosomes has been shown to require G-actin [30].

In HSV-1 infected Hep2 cells, nucleocapsid movement was shown to be sensitive to ATP depletion by sodium azide, decreased temperature, the actin depolymerizing agent latrunculin A and butanedione monoxime (BDM), a chemical inhibitor of myosin motors [31]. These results suggest that herpesvirus nucleocapsids move toward the inner nuclear membrane in a directed and ATP-dependent manner as opposed to free diffusion. Additionally, intranuclear movement was insensitive to cytochalasin D, implying that nucleocapsids may be utilizing non-classical actin that lacks the binding site for this drug. Future work is needed to determine definitively if a myosin motor is trafficking capsids to the nuclear periphery along F-actin, and if so, the specific myosin involved.

As a possible explanation of the mechanism of nucleocapsid intranuclear movement, actin filaments have been shown to form within the nucleoplasm of infected cells. Specifically, serial-section block-face scanning electron microscopy (SBFSEM) and immunofluorescence demonstrated that nuclear F-actin closely associates with capsids in PrV infected neurons [32]. Myosin Va was also found to co-localize with F-actin and GFP-labeled capsids [32]. The direct stimulus for the generation of nuclear actin filaments during herpesvirus infections is unclear. It is possible that these filaments in conjunction with an actin motor (e.g., myosin Va or other processive myosins) provide the ATP-dependent force for nucleocapsid movement.

An electron tomography study of HSV-1 nucleocapsids budding into the perinuclear space revealed 8–19 nm fibers resembling F-actin, which appeared to be connecting the capsid to the primary envelope [33]. It is also possible that these fibers represent tegument proteins, including the nuclear envelopment complex containing pU_L_31/pU_L_34 or proteins associated with them. The nuclear envelopment complex is essential for HSV-1 budding into the perinuclear space [34].

Based on this body of evidence, it seems plausible that herpesvirus nucleocapsids induce nuclear F-actin polymerization and utilize or recruit a myosin motor to traffic capsids to the inner nuclear membrane for primary envelopment.

## 5. Virion Egress and Actin

Late in infection the alphaherpesvirus kinase U_S_3 plays a major role in nuclear egress, but also is responsible for depolymerizaion of actin stress fibers [35–37]. In PrV infections, the U_S_3 kinase causes both actin stress fiber disassembly and the generation of actin-containing cellular extensions in sparsely seeded swine testicle cells [38]. These cellular extensions did not form with a U_S_3-null virus or in cells treated with the actin-stabilizing drug jasplakinolide [38]. These protrusions are important for cell to cell spread, particularly in sparsely seeded cells [38]. The U_S_3 kinase has been shown to bind and phosphorylate (activate) group A p21-activated kinases (PAKs), which are downstream effectors of Cdc42/Rac1 Rho GTPase signaling pathways [39]. In mouse embryo fibroblasts (MEFs), U_S_3-mediated disassembly of actin stress fibers required PAK2 and the formation of cellular projections required PAK1 [39]. Sparsely seeded MEFs derived from PAK1 knock-out mice resulted in reduced PrV spread, but PAK1 was dispensable for viral spread in monolayers, while MEFs derived from PAK2 knock-out mice were impaired for PrV spread in both sparsely seeded and MEF monolayers [39]. It is tempting to speculate that the U_S_3-induced cell extensions may be important primarily when infected cells are not in direct contact with neighboring target cells. The contribution of these extensions for virus spread *in vivo* requires further study.

Actin has been shown to be present in purified herpesviruses. How this incorporation takes place as well as its potential role in infection is unknown. It is possible that the presence of F-actin in virions plays a structural role, since incorporation was enriched in PrV virions lacking tegument components [40–42].

Myosin motors have also been shown to be involved in viral egress. In HSV-1, the tegument protein VP22 has been shown to interact with non-muscle myosin IIA (NMIIA) [43]. Additionally, treatment of Vero cells with the myosin inhibitor BDM at 12 hpi reduced HSV-1 secretion 20-fold and had little effect on the amount of cell-associated virus produced [43]. NMIIA is a non-processive motor that regulates F-actin by cross-linking and contracting actin filaments [44,45]. It has also been shown to be important for fusion pore expansion during exocytosis, which could have implications for virions as they escape from the TGN-derived vesicles and enter the extracellular environment [46]. Recently NMIIA and F-actin, along with Rab6, were shown to be required for the fission of vesicles emerging from the Golgi complex [47]. In cells where NMIIA or Rab6 were inhibited or knocked down, tubules were observed to emanate from the Golgi apparatus representing unfissioned budding vesicles. Protein kinase D (PKD) has been similarly described as required for membrane fission at the TGN [48] and for HSV-1 egress [49]. This presents the possibility that NMIIA, along with Rab6 and PKD, is important for generating vesicles distinct from the Golgi that are free to traffic to the plasma membrane for virion release. Inhibition of NMIIA would prevent these vesicles from severing, but would likely not decrease intracellular infectivity (assuming the unfissioned, emerging tubules are competent for viral budding), consistent with previous observations [43].

Myosin Va (myoVa) is known to be important for secretion of melanosomes [50] and secretory granules [51] by providing transport through cortical F-actin. The transition between microtubule filaments and cortical actin has been described as a “capture” process where the microtubule motor kinesin traffics cargo to the cell periphery where myoVa is required for association of the cargo within the cortical actin and eventually secretion. MyoVa has also been linked to HSV-1 secretion as well as cell surface expression of at least gD, gB and gM [52]. In this study, two red fluorescent protein (RFP)-tagged dominant-negative isoforms (brain and skin) of myoVa were expressed during HSV-1 infection. Secretion of infectivity was down 50–75% compared to control cells expressing RFP alone. Under the same conditions, surface expression of gD, gB and gM were significantly decreased compared to RFP expressing control cells. It is possible that egressing virions within TGN-derived vesicles could behave similarly to other myosin dependent cargo. In this model, kinesin delivers virion-laden vesicles to cortical actin and myoVa transports the vesicles the remaining short distance to the plasma membrane for fusion.

Thus, both NMIIA and myoVa may be involved in functionally distinct and important steps during egress of HSV-1 and likely other herpesviruses. Further work is needed to test the models proposed above and to fully understand the roles of actin and myosin motors during herpesvirus infections.

## 6. Conclusion

**Figure 2.**
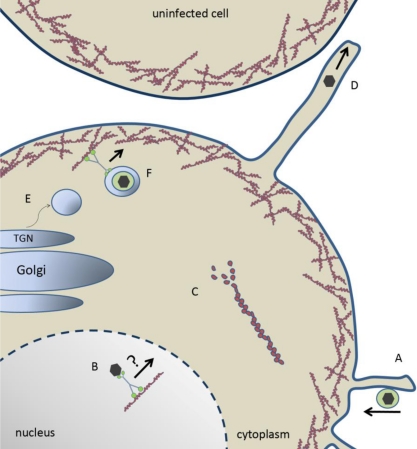
Model of herpesvirus entry, maturation and egress. (**A**) Herpes virion “surfing” towards the plasma membrane along membrane protrusions for entry. (**B**) Capsid trafficking toward the nuclear periphery for budding via a myosin motor on F-actin. (**C**) U_S_3-mediated depolymerization of actin stess fibers. (**D**) U_S_3-mediated generation of membrane projection and cell-to-cell spread of herpesvirus. (**E**) Myosin IIA- and Rab6-dependent fission of nascent vesicles from the Golgi body. (**F**) Enveloped virion within a TGN-derived vesicle trafficking through cortical actin via myosin Va toward the plasma membrane for secretion.

Like all viruses, herpesviruses are obligate, intracellular pathogens relying on host machinery to replicate and spread. Actin plays a critical and diverse role in the cell and in the lifecycle of herpesviruses (some examples are illustrated in Figure 2). In this way, virology research is useful not only for expanding our understanding of how viruses function, but help reveal the intricate biology of our own cells. Many details about the interaction of herpesviruses with actin remain to be investigated. For example, it would be of interest to know what specific viral proteins interact with host myosin motors and how myosin-driven activities are regulated during infection. Nuclear actin is an emerging area of study in virology, as well as cell biology. A better understanding of how nuclear F-actin formation is triggered and how it contributes to viral infection would be of great interest to both fields. Elucidating the mechanism behind the ATP-, F-actin and possibly myosin-dependent movement of nucleocapsids would be an important discovery for nuclear trafficking events. Undoubtedly, much remains to be learned from the interplay of virus and host.
